# Gene Regulation Network Based Analysis Associated with TGF-βeta Stimulation in Lung Adenocarcinoma Cells

**DOI:** 10.15171/ijb.1308

**Published:** 2017-03

**Authors:** Lin Hua, Hong Xia, Wei-ying Zheng, Li An

**Affiliations:** ^1^ School of Biomedical Engineering, Capital Medical University, Beijing, 100069, China; ^2^ Beijing Key Laboratory of Fundamental Research on Biomechanics in Clinical Application, Capital Medical University, Beijing, 100069, China; ^3^ Beijing Institute of Respiratory Medicine, Beijing Chao-Yang Hospital, Capital Medical University, Beijing, 100028, China

**Keywords:** Gene regulation network, TGF-β, Lung adenocarcinoma cell;, Diff erentially expressed genes

## Abstract

**Background:**

Transforming growth factor (TGF)-β is over-expressed in a wide variety of cancers such as lung adenocarcinoma. TGF-β plays a major role in cancer progression through regulating cancer cell proliferation and remodeling of the tumor micro-environment. However, it is still a great challenge to explain the phenotypic effects caused by TGF-β stimulation and the effect of TGF-β stimulation on tumor micro-environment.

**Objectives:**

To address this issue, in the present study we used two time-course microarray data in human lung adenocarcinoma cells and applied bioinformatics methods to explore the gene regulation network responding to TGF-β stimulation in lung adenocarcinoma cells.

**Materials and Methods:**

The time-dependent reverse-engineering method, protein-protein interaction network analyses, and calculation of the similarity measures between the links were used to construct gene regulatory network and to extract gene clusters.

**Results:**

Utilizing the constructed gene regulation network, we predicted NEFL and LUC7A show the opposite and the same change with C21orf90 if HAND2 is knocked-out after treatment with TGF-β_1_ for 4 hours and for 12 hours respectively. FGG and HSPC009 are predicted to display the opposite change with NEFL if CSMD1 is knocked out after treatment with TGF-β_1_ for 12 hours. Additionally, by integrating two datasets, we specially identified several nested clusters which included those genes regulated by TGF-β stimulation in lung adenocarcinoma cells.

**Conclusions:**

Our analysis can help a better understanding regarding how TGF-β stimulation causes the expression change of a number of the genes and provide a novel insight into TGF-β stimulation effect on lung adenocarcinoma cells.

## 1. Background

Transforming growth factor (TGF)-b plays a major role in the initiation and progression of cancers. In the earlier stages, TGF-β works as a tumor suppressor via inhibiting cell growth and apoptotic induction in the epithelial cells (1). On the contrary, in the later stages, the epithelial cells will become refractory to the growth inhibitory effect of TGF-β which acts as a tumor promoter, increasing the tumor-promoting activity, cause the invasiveness, and metastasis (1,2). Several previous observations have approved that the normal epithelial cells show differential response to TGF-β stimulation as compared to the tumor cells (3). In response to TGF-β stimulation, tumor cells display an increased production of proteases and down-regulation of the inhibitors of the proteases, whereas this is not observed in the normal cells (4). Recent studies have found some novel regulation relationships between TGF-β and genes in lung tumor cells. For example, Wang *et al*. have found that TGF-β regulates the proliferation of lung adenocarcinoma cells by inhibiting PIK3K3 expression (5). Yu *et al*. unveiled a novel link between TGF-β and Rac1. They considered the atypical Rac1 activator DOCK4 as a key component of the TGF-β/Smad pathway that promotes lung adenocarcinoma cell extravasation and metastasis (6). Risolino *et al*. have found that the transcription factor PREP1 induces Epithelial–mesenchymal transition (EMT) and metastasis by controlling the TGF-β–SMAD3 pathway in non-small cell lung adenocarcinoma (7). However, it is not clear if various actions of the TGF-β on the normal and lung tumor cells are due to differential gene regulations.

We know that gene regulatory networks have an important role in each life processes including cell differentiation, cell metabolism, cell cycle, and signal transduction (8). For example, De and Berx have claimed that the EMT-associated reprogramming of the cells not only suggests that fundamental changes in several regulatory networks might occur but also that an intimate interplay exists between such networks. Disturbance of the controlled epithelial cells’ reproduction balance is triggered by altering several layers of regulation (9). Meng *et al*. constructed the lung adenocarcinoma related regulatory network using microarray data and found that FLI and TAL1 promote TGFBR and KDR expression respectively; the result of which is activation of the TGF-β signaling pathway (10). Genovese *et al*. uncovered a novel regulation of TGF-β signaling via a Smad4 transcriptomic network by miR-34a through constructing a network model based on the complex multidimensional cancer genomic data (11). Vilar *et al*. developed a concise computational model of the TGF-β pathway and showed that the first layer of communication with the environment, the ligand-receptor network, is not merely a passive transducer of the signals but rather embeds properties that make it a signal processing unit (12). Therefore, these evidence support the identification of the intricate interplay between genes responsible for the observed phenotypes based on the gene regulation network, and will help to understand how TGF-β stimulation affects the biological change of normal cells or tumor cells.

## 2. Objectives

In the present study, we used two time-course microarray data in human normal lung epithelial cell and lung adenocarcinoma cell and applied novel bioinformatics methods to explore the gene regulation networks associated with TGF-β stimulation in two different cell lines. We predicted the network change when several genes regulated by TGF-β are knocked-out. Our analysis can help to understand better how TGF-β causes the expression change of other genes and gives an insight into TGF-β effect on lung adenocarcinoma cells, as well as the development of the more effective lung adenocarcinoma treatment strategies.

## 3. Materials and Methods

### 3.1. TGF-β Regulated Gene Profiling in the Normal Lung Epithelial Cells and Lung Carcinoma Cells

#### 3.1.1. Data Description

To explore the network change when genes regulated by TGF-β are knocked-out, we selected the gene expression profiling regulated by TGF-β in the normal lung epithelial cells (HPL1D) and lung carcinoma cells (A549). HPL1D and A549 cells were treated with TGF-β_1_ for 1, 4, and 12 hours, the total RNAs were extracted and were used for microarray using human 19 k arrays (4). This dataset was downloaded from Gene Expression Omnibus database (http://www.ncbi.nlm.nih.gov/geo/) (accession No. GSE7436). Genes which show log 2 ratios greater than 0.37 (1.3 fold induced) or less than -1.5 (0.3 fold induced) at any one of the time points are considered as up-regulated by TGF-β or down-regulated by TGF-β, respectively. To help observe the data distribution, we used the supra-hexagonal map (13) to display the samples’ characteristics. The supra-hexagonal map provides a choice for calculating the covariance matrix based on a variety of different distance metrics. It converts the gene-sample matrix into the codebook matrix and genes with similar data patterns were taken as the same or nearby nodes in the map by applying a self-organizing learning algorithm for the symmetric topology of the supra-hexagonal map. We observed the obvious gene expression difference between normal lung epithelial cells (HPL1D) and lung carcinoma cells (A549) at different time points (supplementary Fig. 1).

#### 3.1.2. Differentially Expressed Genes Filtration under Different Time Points

In this analysis, we used R-bioconductor limma package (http://www.bioconductor.org) to select differentially expressed genes under different time points between normal lung epithelial cells (HPL1D) and lung carcinoma cells (A549). To select those genes that significantly distinguish the normal lung epithelial cells from the lung carcinoma cells as well as avoiding the constructed network complication which may cause the network can not be well explained; therefore, we kept the top 20 most significant differentially expressed genes distinguishing HPL1D from A549 at each time point. In addition, we also selected the top 20 ranked genes with the global differentially expression across all time points (Supplementary Table 1). Finally, all of the reserved differentially expressed genes were used for further network construction.

#### 3.1.3. Construction of the Gene Regulation Network

In the present study, we used the time-dependent reverse-engineering method to construct gene regulatory network. This method relies on a Lasso penalized estimation of the linear regression model (14). Suppose the linear regression model is:

(1) $$y_{i}=\sum_{j=1}^{p}\beta_{j}x_{j}+\eta_{i}(i=1,2,...,N)$$

Where *_i_* is the response, while *x_i_ =(x_il_,...,x_P_)^T^* are the predictors, and *η_i_* is a noise following some probabilistic distributions. Assume that the predictors are standardized and that the response is centered, the Lasso penalized estimation is then given by:

(2) $$\hat{\beta }(\lambda )=\underset{\beta \in R^{p}}{\arg\min}[{\sum_{i=1}^{N}\left(y_{i}-\sum_{j=1}^{p}\beta_{j}x_{j}\right)}^{2}+\lambda {\|\beta \|}_{1}]$$

Where *λ* is a non-negative scalar that determines the level of the constraints. Based on this method, the time dependent reverse-engineering method for constructing gene regulation network was described as followings simply (14):

Suppose that we have selected *N* genes across *T* time points and for *P* subjects; we define *x_npt_*is the expression of gene* n* for individual *p* at the time-point *t*. Since each gene is considered as a response variable, therefore the model is composed of *N* linear regression models. It is defined:

 (3) $${\tilde{X}}_{n..}=\left(\frac{{\tilde{X}}_{n1.}}{{\tilde{X}}_{nP.}}\right),{\hat{X}}_{n..}=\left(\frac{{\hat{X}}_{n1.}}{{\hat{X}}_{nP.}}\right)$$

Where

 (4) $${\tilde{X}}_{ni.}=\left(\frac{{\tilde{X}}_{nit\;2}}{{\tilde{X}}_{nit\;T}}\right),{\hat{X}}_{ni.}=\left(\frac{{\hat{X}}_{nit1}}{{\hat{X}}_{nit\;T-1}}\right),i=1,2,....P$$

*x_nit_* is the expression of gene *n* for individual *i* at time-point $t.{\tilde{x}}_{n..}$ is the regulated gene and ${\tilde{x}}_{n^{\prime }..}(n^{\prime }=1,2,...N)$ are the regulators. The linear regression model is described as the following equation:

(5) $${\tilde{x}}_{n..}=\sum_{n^{\prime }=1}^{N}F_{m(n^{\prime })m(n)}w_{n^{\prime }n}{\hat{x}}_{n^{\prime }..}+\varepsilon_{n}$$

Where *m(n) * is the function that maps gene *n* to its time-cluster and *F_m(n')m(n)_* is a *T-1* square matrix that describes the action of the genes. *w_n'n_* is the strength of the connection from gene* i* toward gene *j*. ε is a noise vector of length *T-1* with *E(ε)* =0 and *Var(ε) = σ^2^* . The Lasso estimate for linear regression is obtained with the following formula:

(6) $$(\hat{w},\hat{F})=\underset{\begin{array}{l} w_{n^{\prime }n}\in R,1\leq n^{\prime },n\leq N\\ F_{b}\in M_{T-1}(R.)1\leq a,b\leq T \end{array}}{\arg\min}[\sum_{n=1}^{N}{\left({\tilde{x}}_{n.}-\sum_{n^{\prime }=1}^{N}F_{m(n^{\prime })m(n)w_{n^{\prime }n}}{\hat{x}}_{n^{\prime }..}\right)}^{2}]$$

Where $n=1,2,...N,\sum_{n^{\prime }=1}^{N}w_{n^{\prime }n}\leq \lambda_{n}.\lambda_{n}$. is a non-negative scalar that determines the level of the constraints. At each step, the estimation of matrices *F_m(n')m(n)_* is done several times throughout the cross-validation. In this analysis, the reserved top ranked differentially expressed genes were used to construct gene regulation network. The Cascade package (15) of R software (http://www.r-project.org) was used to implement this program.

### 3.2. TGF-β Regulated Gene Expression Profiling in the Lung Carcinoma Cells

#### 3.2.1. Data Description

To further explore the gene regulation relationships associated with TGF-β stimulation in the lung carcinoma cells, we performed the additional analysis for another time course microarray data. We downloaded this dataset from Gene Expression Omnibus (accession No. GSE17708 (16)). This dataset was obtained from human A549 lung adenocarcinoma cells undergoing TGF-β-induced epithelial-mesenchymal transition (EMT). A549 lung adenocarcinoma cell line was treated with 5 ng.mL^-1^ TGF-β for 0, 0.5, 1, 2, 4, 8, 16, 24, and 72 hours to induce EMT. Samples were assayed using Affymetrix HG_U133_plus_2 arrays with 54,675 probe-sets.

#### 3.2.2. Filtration of Differentially Expressed Genes at Different Time Points

For GSE17708 dataset, at each time point, excluding 0 hours, the differentially expressed genes were selected for their ability that distinguishes the expression at this time point from the expression at 0 hour. In the previous analysis, the genes with p-value <0.001 and >2-fold change at each time point were considered as differentially expressed genes (17). We kept these genes for further analysis, as well.

### 3.3. TGF-β Associated Gene Clusters Identification in Lung Adenocarcinoma Cells; an Integration of the Two Datasets

To understand the functional gene clusters (i.e., networks) associated with TGF-β in lung adenocarcinoma cells, we mapped the differentially expressed genes extracted from the two datasets; A)- the filtered differentially genes for GSE7436 and B)- the filtered differentially expressed genes for GSE17708, to protein–protein interaction (PPI) networks using the STRING database (http://string-db.org) which is a database of known and predicted protein interactions. To avoid those pairs with a lower association to the lung adenocarcinoma into the analysis, we filtered the gene pairs which only include those genes related to the lung cancer using Online Mendelian Inheritance in Man (OMIM) database (18). In the current study, we consider a gene network to be a set of closely interrelated links. By calculating the similarity measures between links, we can determine the expected amount of overlap clusters around a gene. A gene belongs to multiple clusters means that this gene is important in the gene regulation network. The program was implemented with Linkcomm package (19) of R software (http://www.r-project.org).

## 4. Results

### 4.1. TGF-β Regulated Gene Expression Profiling in Normal Lung Epithelial Cells and Lung Carcinoma Cells (for GSE7436 Dataset)

#### 4.1.1. Extracting the Significant Differentially Expressed Gene

**Figure 1:**
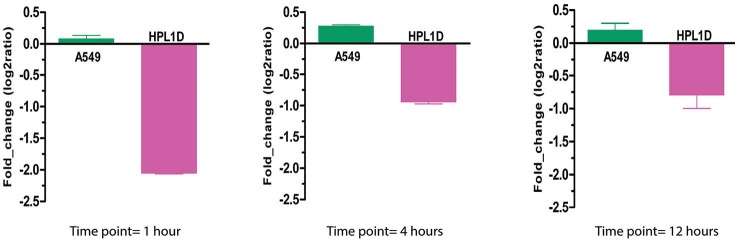
The Fold_change (log2ratio) of the HAND2 at three-time points. HAND2 displayed an obvious up-regulation trend (log2ratio>0) in the lung carcinoma cell (A549) and down-regulation trend (log2ratio<0) in the normal lung epithelial cells (HPL1D).

For each time point, we selected the top 20 most significant differentially expressed genes between normal lung epithelial cells and lung carcinoma cells. We also kept the top 20 ranked genes with the global differentially expression. Finally, the total of 80 differentially genes was identified. Among the global differentially expressed genes, HAND2 displayed the obvious down-regulation in the normal lung epithelial cells after treatment with TGF-β_1_ for 1 hour (Fig. 1). Although HAND2 did not display an obvious up- or down-regulation in the lung carcinoma cell at three-time points, the trend of up-regulation seems obvious (log2ratio>0). There are some studies that have demonstrated HAND2 over-expression in the lung squamous cell carcinomas and de-regulated in the histological subtypes (4). Recent evidence has suggested that HAND2 methylation is a common and crucial molecular alteration in several types of cancers, and could be employed as a potential biomarker for the early detection as well as a predictor of the treatment response (20).

#### 4.1.2. Construction of the Gene Regulation Network Associated with TGF-β Stimulation

We used the identified 80 differentially expressed genes to construct gene regulation network associated with the TGF-β stimulation. Considering that a larger number of edges in the network makes it difficult to interpret the relationships between genes, therefore, we selected a cutoff to simplify the network. In order to choose the best cutoff, we used the evolution method which allows us to see the evolution of the network when the cut off is growing up. At each step, the positions of the genes are re-calculated. The current choice of cutoff relies on a p-value which corresponds to the adequacy of the data to a power law distribution. The p-value should ensure the scale-freeness of the network is reliable (14,21). Finally, a cutoff of 0.10 was selected to filter the network (supplementary Fig. 2). The filtered network is shown in the supplementary Figure 3.

#### 4.1.3. Prediction of Gene Expression Modulations after a Knocked-out Experiment

**Figure 2:**
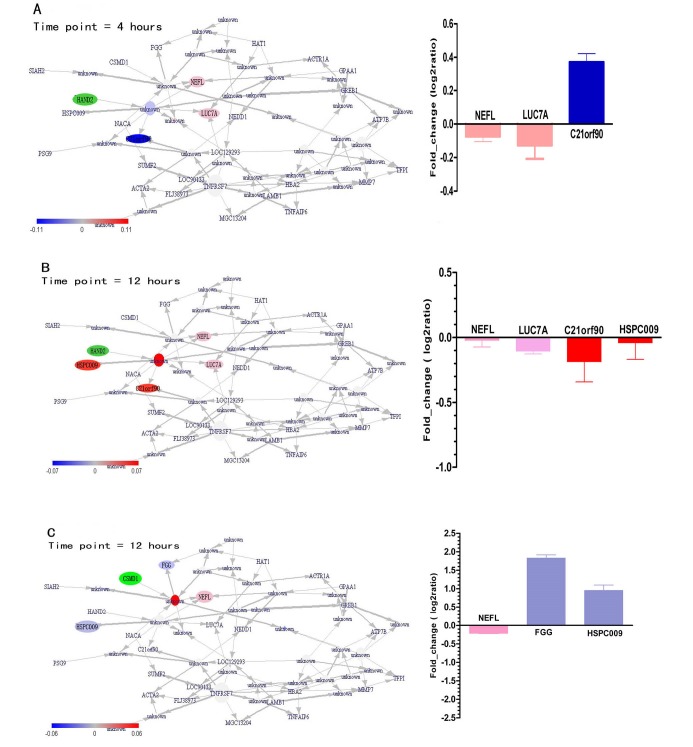
The changed gene expressions when special genes are knocked out after treatment with TGF-βeta1. (A) The changed genes’ regulations when HAND2 is knocked out after treatment with TGF-β_1_ for 4 hours. When HAND2 (colored by green) is knocked out, NEFL and LUC7A (colored by pink) show the opposite change trend with C21orf90 (colored by deep blue). Meanwhile, C21orf90 is up-regulated (log2ratio>0.37). (B) The changed gene expressions when HAND2 is knocked out following to the treatment with TGF-β_1_ for 12 hours. When HAND2 (colored by green) is knocked out, NEFL (colored by pink), LUC7A (colored by pink), C21or f90 (colored by red), and HSPC009 (colored by red) display the same changed trend. (C) The changed gene expression when CSMD1 is knocked out after treatment with TGF-β_1_ for 12 hours. When CSMD1 (colored by green) is knocked out, FGG and HSPC009 (colored by purple) have displayed the opposite change with NEFL (colored by pink). Meanwhile, FGG and HSPC009 are all up-regulated markedly (log2ratio>0.37).

Next, we wanted to predict the changed genes’ regulations if expressions of some genes are knocked out. We found when HAND2 is knocked-out following to the 4 hours treatment with TGF-β_1_, NEFL, and LUC7A show the opposite change trend with C21orf90 which is up-regulated (Fig. 2A). When HAND2 is knocked out after treatment with TGF-β_1_ for 12 hours, NEFL, LUC7A, C21orf90, and HSPC009 displayed the same change trend (Fig. 2B). Meanwhile, we found C21orf90 to show a different expression trend after knocking out of HAND2 at different time points. Although few studies have confirmed the direct association between C21orf90 and lung carcinoma, the expression of C21orf90 was approved to be related to the lung parenchyma which is affected by the abnormal inflammatory immune response (22). When CSMD1 was knocked out following to 12 hours of TGF-β_1_, FGG and HSPC009 displayed the opposite change compared to the NEFL. FGG and HSPC009 were all up-regulated noticeably (Fig. 2C). We know that CSMD1 is a tumor suppressor, and a previous array-based comparative genomic hybridization (aCGH) analysis detected the loss of CSMD1 in lung squamous cell carcinomas (23). From the analysis, we can observe that knocking-down of CSMD1 causes the change in the FGG expression. Although the current evidence can not support the regulation relationships between CSMD1 and FGG, it has been reported that the expression of FGG changed during EMT of lung cancer by several genes such as FOXA1 knockdown in A549 cells (24). Therefore, these potential findings need to be validated by more molecular biology experiments in future studies.

### 4.2. Identification of Gene Clusters Associated with TGF-β in Lung Adenocarcinoma Cells (Integration of Two Datasets)

After 2,714 differentially expressed genes which include 80 differentially expressed genes extracted from GSE7436 and 2,634 differentially expressed genes extracted from GSE17708 were mapped to the protein–protein interaction network, 273 gene pairs which also include genes related to the lung cancer were filtered. Then we used Jaccard coefficient to cluster links between these genes. When cutting the dendrogram at a point (height = 0.5972) where the partition density is maximized, 17 gene clusters were identified (supplementary Fig. 4). Meanwhile, the number of nodes (genes) in the largest gene cluster is 19.

**Figure 3:**
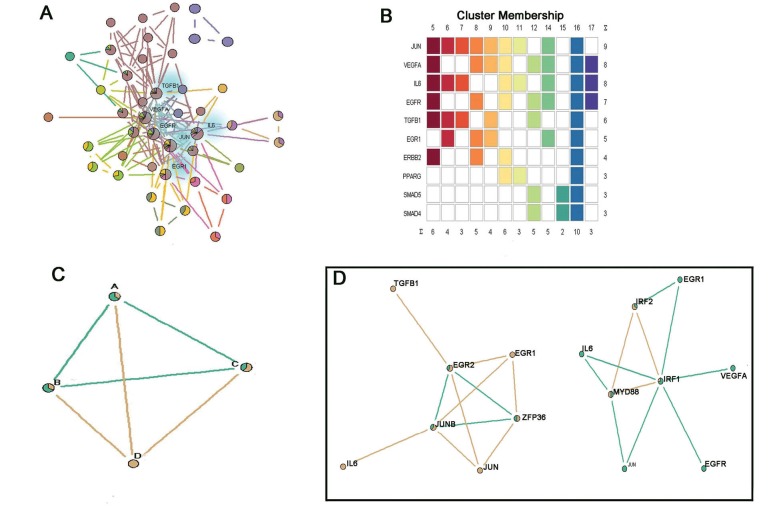
Visualization of the linked clusters. (A) Node pies graph. The fraction of the total number of edges that a gene has in each cluster is depicted using a pie chart. (B) The cluster membership for genes that belong to the most clusters. Colors indicate cluster-specific membership. Each color represents a gene cluster. For example, JUN, VEGFA, IL6, EGFR, TGFB1, and ERBB2 belong to the fi fth gene cluster. (C) The graph defi nition for the nested clusters of genes. (D) Visualization of the two nested gene cluster structures.

We observed 6 genes that belong to more than five gene clusters (Fig. 3A and Fig. 3B), including JUN (belongs to 9 clusters), VEGFA (belongs to 8 clusters), IL6 (belongs to 8 clusters), EGFR (belongs to 7 clusters), TGFB1 (belongs to 6 clusters), and EGR1 (belongs to 5 clusters). It is noted that these genes were all associated with the TGF-β induction and activity in the lung cancer. For example, it has been reported that TGF-β is the major inducer of the interleukin-6 (IL-6) and vascular endothelial growth factor (VEGF), and the increased production of TGF-β is followed by the increased IL-6 and VEGF secretion related to tumor cell proliferation (25). In addition, Finocchiaro *et al*. have suggested the role of TGFB as a mediator of the intrinsic resistance to EGFR tyrosine kinase inhibitors in non-small cell lung cancer (NSCLC) patients (26). As well, genes that belong to multiple gene clusters are possible to be discovered in the sets of genes that belong to clusters that are entirely nested within a larger cluster of the genes. For example, gene A, B C involved in a cluster (green color) are entirely nested within the larger cluster including gene A, B, C and D (brown color) (Fig. 3C). Therefore, genes involved in those nested clusters should be noted for their potential interaction effect with other genes. In the present study, we acquired some nested gene clusters. For example, Figure 3D shows the genes involved in the two nested gene clusters. We found that JUNB, EGR2, ZFP36, IRF1, IRF2, and MYD88 all are involved in the nested gene clusters.

### 4.3. Gene Set Enrichment Analysis (Integration of Two Datasets)

**Figure 4:**
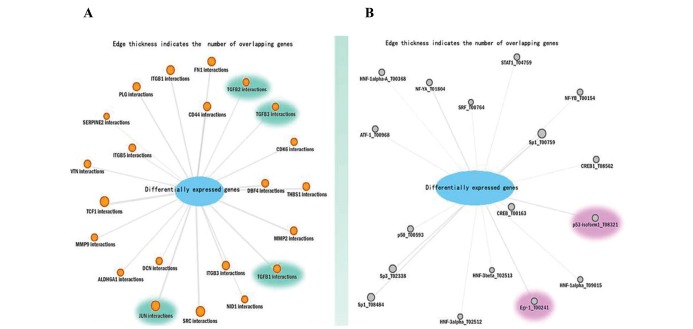
Gene set enrichment analysis for 2,714 diff erentially expressed genes extracted from the two datasets using ConceptGen software. (A) Protein interactions among the diff erentially expressed genes treated with TGF-β induction. (B) Transcription factors’ interactions among the diff erentially expressed genes treated by TGF-β induction.

To explore the potential biological knowledge of the identified gene sets or gene networks, the total of 2,714 differentially expressed genes extracted from the two datasets were uploaded to ConceptGen software (16) which offers over 20,000 concepts comprising 14 different types of biological knowledge for performing the enrichment analysis. The significance of the over-representation is measured by a modified Fisher’s exact test (p-value) and q-values which take into account the estimated proportion of false positives incurred based on p-values. By default, only concepts with q-values < 0.05 are displayed in this analysis. The results can partly reflect TGF-β induction and the complexity of EMT process. For example, the protein interactions among the differentially expressed genes link the TGFB1, TGFB2, and TGFB3 interactions directly (Fig. 4A). Moreover, we found these differentially expressed genes set also links JUN interactions. It has been reported that dysregulated c-jun expression may be involved in the acquisition of anchorage independence in the process of human lung carcinogenesis (27). Transcription factors interactions among the differentially expressed genes link the transcription factor Egr-1 and p53 (Fig. 4B). Previous studies have found that Egr-1 mediates the stimulation of collagen transcription elicited by TGF-β. Also, it was found that TGF-β is necessary for the development of the pulmonary fibrosis (28). It has been reported that TGF-β causes a time- and dose-dependent increase in Egr-1 protein and mRNA levels as well as enhancement of the Egr-1 gene transcription via serum response elements in the normal fibroblasts (29). In addition, recent studies have shown that p53 affects TGF-β /SMAD3-mediated signaling, cell migration, and tumorigenesis. Mutant p53 proteins also regulate Nox4-dependent signaling in TGF-β-mediated cell motility (30). Other enrichment analyses such as those carried out based on Gene Ontology (GO) or Kyoto Encyclopedia of Genes and Genomes (KEGG) have also resulted in finding that these differentially expressed genes set are related to the TGF-β induction and activities.

In summary, we observed NEFL and LUC7A show an opposite and the same change with C21orf90 if HAND2 is knocked-out following to TGF-β_1_ treatment for 4 and 12 hours, respectively. In addition, FGG and HSPC009 display an opposite change with respect to NEFL if CSMD1 is knocked out after treatment with TGF-β_1_ for 12 hours. Furthermore, by integrating the two datasets, we specifically have identified several nested clusters which include those genes regulated by the TGF-β in the lung adenocarcinoma cells.

## 5. Discussion

It is known that TGF-β plays important roles in cancer progression, affecting both tumor and stromal cells. Gene regulatory networks may have a potential influence on cell differentiation and cell metabolism. Most influence of the TGF-β is brought about by regulation of gene expression. In the current study, our aim was to explore the intricate interplay between genes in response to the TGF-β stimulation in the lung adenocarcinoma cells. Our analysis shows that when a number of genes involved in gene regulation network are knocked out, several other genes will show up-regulation or down-regulation trends. By integrating two time-course microarray data in human lung adenocarcinoma cells, we specifically have identified some nested gene clusters and found that TGFB1, EGR1, EGR2, EGFR, IL6, JUN, and JUNB all are involved in these clusters. The previous evidence has confirmed the potential regulation role of TGFB on these genes, such as EGR1, EGFR, and IL6 in the lung cancer. Taken together, our analysis can help to understand better how TGF-β causes changes in the expression of several other genes and gives an insight into TGF-β effect on lung adenocarcinoma cells.

It should point out the limitation of our study. The shortcoming of time-course microarray data is the low sample size. Although we integrated two datasets to implement the analysis in this paper, however, this integration is not enough. In the practice, utilizing more available time-course datasets and performing the synthesized analysis such as Meta-evaluation will help to improve the reliability of the results. Moreover, our prediction for the expression change of the NEFL, LUC7A, C21orf90, FGG, and HSPC009 if HAND2 and CSMD1 are knocked-out TGF-β_1_ treatment at different time points need to be validated by further molecular biology experiments in the future studies.

## Acknowledgements

This work is supported by the Beijing Natural Science Foundation (Grant Nos. 7142015), and National Science Foundation of China (Grant Nos. 31100905). This study is also funded by the foundation-clinical cooperation project of the Capital Medical University (14JL43, 16JL58 and 17JL54).

## Supplementary File

Supplementary File
