# Supplementary material for: Gene Regulation Network Based Analysis Associated with TGF-βeta Stimulation in Lung Adenocarcinoma Cells
Source: Iran J Biotechnol. 2017 Mar;15(1):1–9. doi: 10.15171/ijb.1308 (PMC5582248; doi:10.15171/ijb.1308)
Supplement: Supplementary File [file ijb-15-1-s001.pdf]

Supplementary Table 1

The Top 20 differentially expressed genes at different time point according to their significance

Rank	1 hour	4 hours	12 hours	Global across all time points
1	FLJ23861	unknown	ACTA2	HAND2
2	MMRN	MGC13204	HSPC009	DDEF2
3	RAD21	GREB1	ACTR1A	SFRS15
4	unknown	unknown	unknown	Sez6
5	GPAA1	unknown	MMP7	unknown
6	unknown	LOC129293	unknown	TRIM29
7	HAT1	unknown	unknown	MYD88
8	unknown	unknown	C21orf90	SLC2A1
9	TNFRSF7	unknown	LUC7A	PSG9
10	unknown	unknown	NEDD1	RAB9B
11	unknown	unknown	ATP7B	unknown
12	unknown	unknown	NEFL	SIAH2
13	NACA	unknown	TFPI	unknown
14	unknown	unknown	unknown	IRF2
15	LAMB1	unknown	TNFAIP6	unknown
16	unknown	HBA2	FGG	unknown
17	unknown	unknown	unknown	MRPS6
18	SMC4L1	SUMF2	unknown	CSMD1
19	unknown	FLJ38973	unknown	unknown
20	unknown	unknown	LOC90133	HKR2

Note: unknown indicates the gene symbol was not identified in the dataset.

Supplementary Figure 1

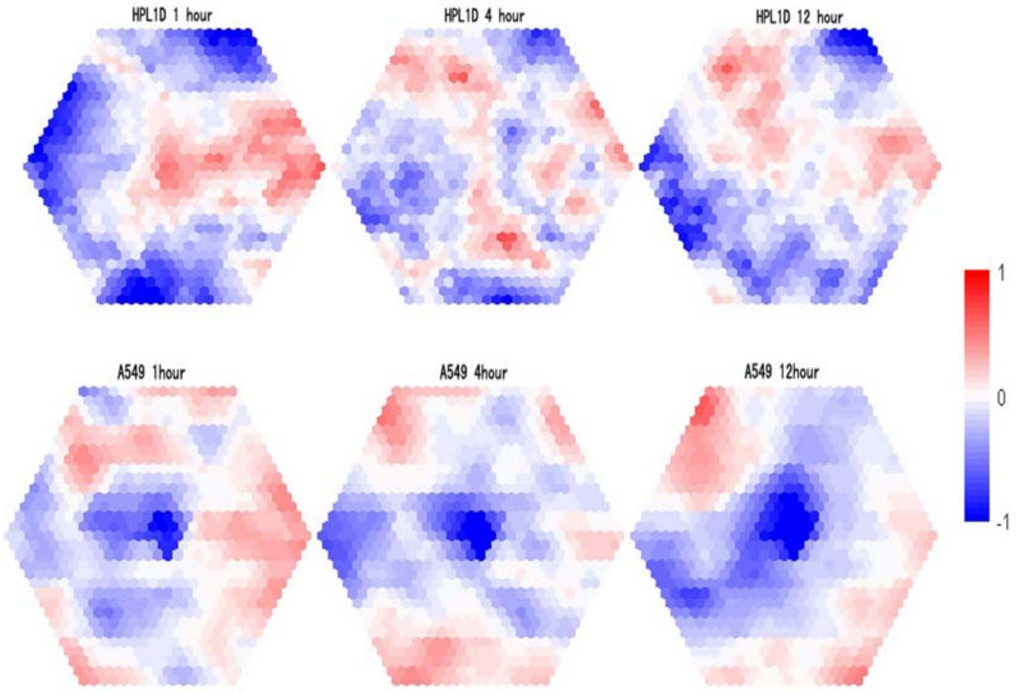

Supplementary Figure 1. Supra-hexagonal maps to visualize samples. The input data was converted into the codebook matrix, and genes with the same or similar activity patterns are spatially clustered to the same or nearby map nodes. This graph displays the gene expression distribution in normal lung epithelial cells (HPL1D) and lung carcinoma cells (A549) treated with TGF-β for 1, 4 and 12 hours respectively.

Supplementary Figure 2

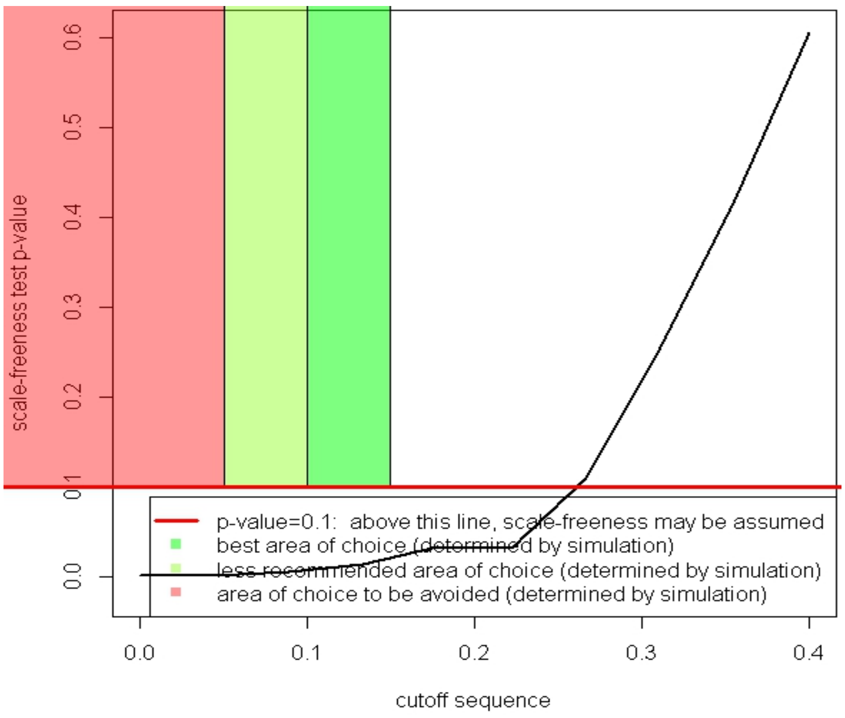

Supplementary Figure 2. Evolution of scale-freeness of the network in function of the cutoff. The p-value corresponds to the adequacy of the data to a power law distribution.

Supplementary Figure 3

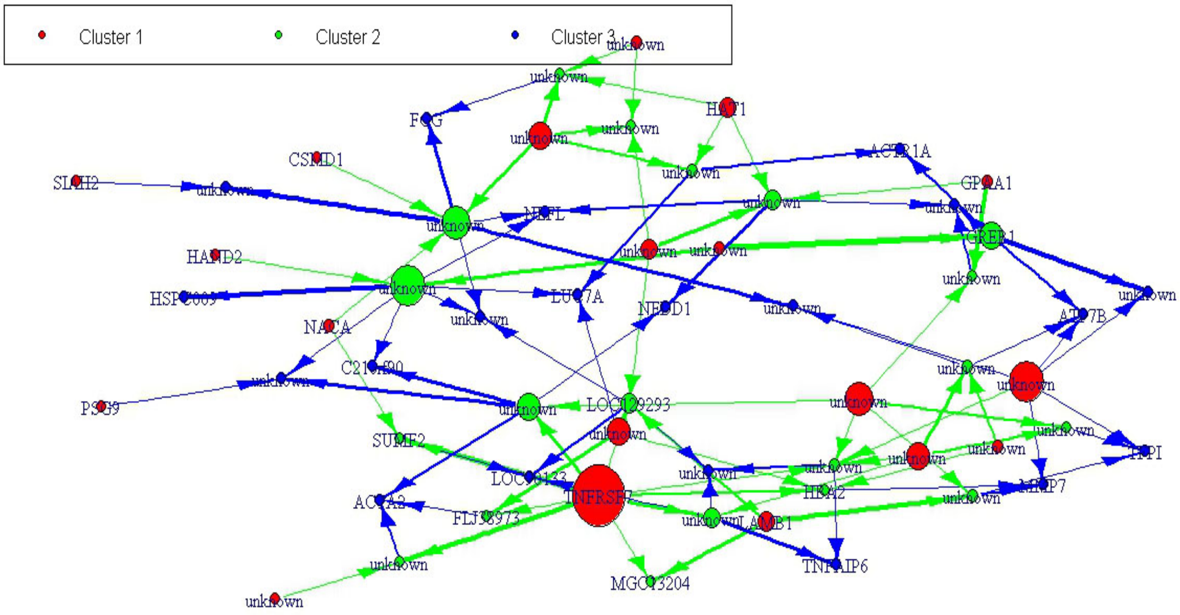

Supplementary Figure 3. The simplified gene regulation network associated with TGF-β. This network is an evolution network with the cut off of 0.10.

Supplementary Figure 4

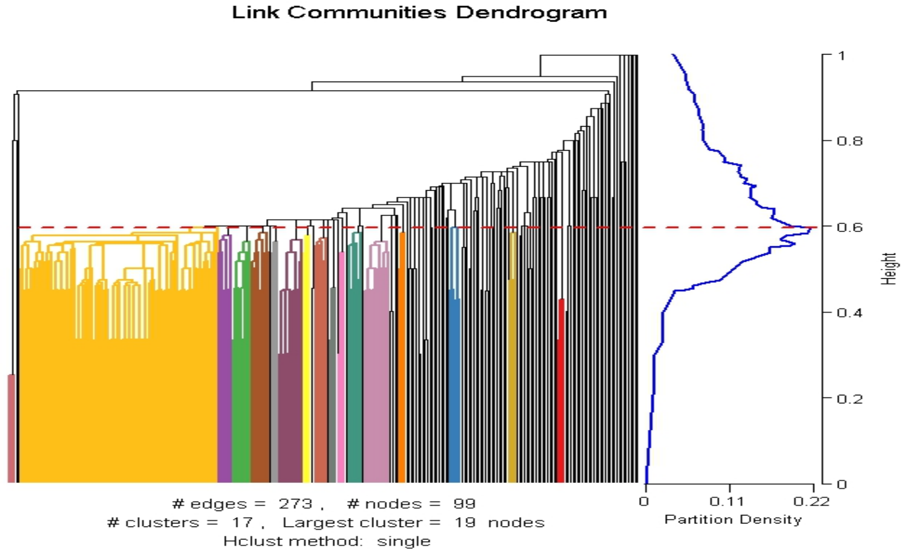

Supplementary Figure 4. The gene communities that result from cutting the dendrogram at a point (Height=0.5972).
